# Supplementary material for: Body fat percentage is independently associated with lower pulmonary function in Korean never-smokers: A cross-sectional analysis of 33,748 adults
Source: PLoS One. 2026 Feb 27;21(2):e0341918. doi: 10.1371/journal.pone.0341918 (PMC12948139; doi:10.1371/journal.pone.0341918)
Supplement: S1 Fig — (PDF) [file pone.0341918.s001.pdf]

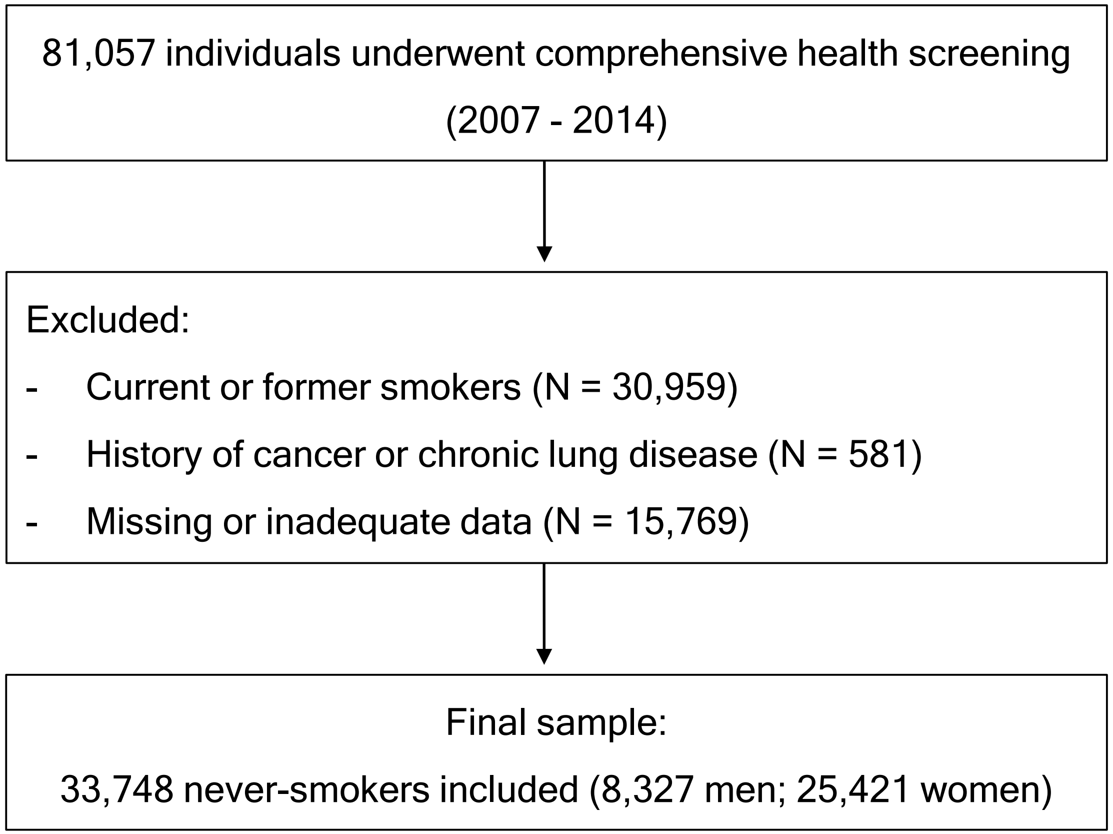
81,057 individuals underwent comprehensive health screening (2007 - 2014)
Excluded:
- Current or former smokers (N = 30,959)
- History of cancer or chronic lung disease (N = 581)
- Missing or inadequate data (N = 15,769)
Final sample:
33,748 never-smokers included (8,327 men; 25,421 women)
