# Supplementary material for: Body fat percentage is independently associated with lower pulmonary function in Korean never-smokers: A cross-sectional analysis of 33,748 adults
Source: PLoS One. 2026 Feb 27;21(2):e0341918. doi: 10.1371/journal.pone.0341918 (PMC12948139; doi:10.1371/journal.pone.0341918)
Supplement: S1 Table — (DOCX) [file pone.0341918.s002.docx]

**Supplementary Table 1. Prevalence of LLN-defined spirometric impairment by body fat percentage quartile, stratified by sex**

|  |  | Body fat percentage quartiles | | | |  |
| --- | --- | --- | --- | --- | --- | --- |
| Variable | Total | Q1 (lowest) | Q2 | Q3 | Q4 (highest) | *P* for trend |
| Men (n = 8,327) |  |  |  |  |  |  |
| FVC% < LLN | 768 (9.2%) | 195 (9.4%) | 163 (7.8%) | 170 (8.2%) | 240 (11.5%) | < 0.001 |
| FEV_1_% < LLN | 250 (3.0%) | 41 (2.0%) | 57 (2.7%) | 63 (3.0%) | 89 (4.3%) | < 0.001 |
| FEV_1_/FVC ratio < LLN | 314 (3.8%) | 70 (3.4%) | 80 (3.8%) | 83 (4.0%) | 81 (3.9%) | 0.720 |
| Restrictive spirometric pattern | 725 (8.7%) | 187 (9.0%) | 152 (7.3%) | 160 (7.7%) | 226 (10.9%) | < 0.001 |
| Women (n = 25,421) |  |  |  |  |  |  |
| FVC% < LLN | 3,101 (12.2%) | 1,082 (17.0%) | 863 (13.6%) | 639 (10.1%) | 517 (8.1%) | < 0.001 |
| FEV_1_% < LLN | 698 (2.7%) | 195 (3.1%) | 189 (3.0%) | 157 (2.5%) | 157 (2.5%) | 0.062 |
| FEV_1_/FVC ratio < LLN | 852 (3.4%) | 208 (3.3%) | 233 (3.7%) | 227 (3.6%) | 184 (2.9%) | 0.069 |
| Restrictive spirometric pattern | 3,005 (11.8%) | 1,067 (16.8%) | 834 (13.1%) | 617 (9.7%) | 487 (7.7%) | < 0.001 |

Data are presented as n (%) for categorical variables. *P* for trend was calculated across BF% quartiles within each sex.

BF% quartile cut-offs: Men: Q1 ≤ 19.3%, Q2 19.4–22.3%, Q3 22.4–25.5%, Q4 > 25.5%; Women: Q1 ≤ 25.7%, Q2 25.8–29.2%, Q3 29.3–32.5%, Q4 > 32.5%.

LLN for the FEV₁/FVC ratio was calculated for each participant using reference equations. LLN thresholds for FVC% and FEV₁% were 82% and 81%, respectively, based on a previous large-scale Korean study. Airflow limitation (obstructive impairment) was defined as FEV₁/FVC ratio < LLN. A restrictive spirometric pattern was defined as FVC% < LLN with FEV₁/FVC ratio ≥ LLN. Abbreviations: BF%, body fat percentage; FEV₁, forced expiratory volume in 1 second; FVC, forced vital capacity; LLN, lower limit of normal.
