# Supplementary material for: Body fat percentage is independently associated with lower pulmonary function in Korean never-smokers: A cross-sectional analysis of 33,748 adults
Source: PLoS One. 2026 Feb 27;21(2):e0341918. doi: 10.1371/journal.pone.0341918 (PMC12948139; doi:10.1371/journal.pone.0341918)
Supplement: Attachment — Submitted filename: Response to Reviewers.docx [file pone.0341918.s003.docx]

**Response to Reviewers**

We sincerely thank the reviewers for their careful evaluation and constructive comments, which have substantially improved the clarity, rigor, and interpretability of our manuscript. Below, we provide a detailed, point-by-point response outlining how each comment has been addressed in the revised version.

**Comments by Reviewer #1**

**Comment 1:** Definition of lung function impairment. The authors must be more precise in the definitions. They used % pred for FEV1 and FVC and a standard cut-off for FEV1/FVC ratio. The authors could perform a sensitivity analysis based on LLN. Furthermore, besides flow limitation, the authors can consider the presence of restrictive spirometry.

***Response:*** We sincerely thank the reviewer for this important and constructive suggestion. In response, we performed additional sensitivity analyses using lower limit of normal (LLN)–based criteria to refine the definition of spirometric impairment.

For the FEV₁/FVC ratio, individualized LLN values were calculated for each participant using reference equations, enabling subject-specific classification of airflow obstruction. However, population-specific reference equations for deriving individualized LLN values for FEV₁% and FVC% are currently unavailable for Korean adults. Therefore, we adopted LLN threshold values for FEV₁% (81%) and FVC% (82%) from a previously published large-scale Korean population study, which has been used as a practical surrogate in Korean epidemiologic research.

Using these LLN-based criteria, obstructive and restrictive spirometric patterns were operationally defined and incorporated into the revised analyses. The Results section has been updated to present LLN-based prevalence estimates and multivariable logistic regression analyses, and impaired lung function is now reported in dedicated tables (Table 5 and Supplementary Table 1). The Discussion and Conclusion have been revised accordingly, demonstrating that elevated BF% was selectively associated with restrictive, but not obstructive, spirometric impairment in both men and women. We have also clearly described this hybrid LLN-based approach in the Methods section and acknowledged it as a methodological limitation.

Relevant revisions were made in the Methods (page 7), Results (page 19-21), and Discussion (page 22-25).

**Comment 2:** Discussion. The authors formulate standard principles about the role of BF but do not bring these principles in relation to their findings. For example, BF as endocrine organ: what about the highest lung function impairment in women with lowest BF quartile?.
***Response:*** We appreciate this insightful comment. In the initial version of the manuscript, women appeared to show a different pattern from men, with a higher crude prevalence of restrictive spirometric patterns in the lowest BF% quartile. However, through the additional LLN-based analyses and comprehensive multivariable adjustment requested by the reviewer, we identified that this apparent sex difference was largely attributable to confounding factors.

Specifically, the crude inverse association in women was driven by age, frailty, and reduced fat-free mass, which were more prevalent in women with lower BF% and are independently associated with reduced FVC. After adjustment for these factors, the association was fully reversed, revealing that higher BF% was independently associated with restrictive impairment in women as well, with a magnitude comparable to that observed in men. Accordingly, the revised manuscript no longer concludes that women differ fundamentally from men in this respect.

In the revised Discussion, we now explicitly explain this reversal and emphasize that the adverse pulmonary effects of BF% are predominantly mechanical rather than endocrine-dominant, and are consistent across sexes once confounding is appropriately addressed. This clarification improves internal consistency between the Results and Discussion and aligns interpretation with the final adjusted analyses.

These revisions are detailed in the Discussion section (page 23-24).

**Comment 3:** A major limitation of this retrospective study is the absence of lung volume measurements. The authors must address this limitation.

***Response:*** We thank the reviewer for highlighting this important limitation. We now explicitly acknowledge that total lung capacity and residual volume were not measured, and therefore restrictive spirometric patterns identified in this study may reflect obesity-related mechanical limitation rather than intrinsic restrictive lung disease. This limitation has been clearly stated in the Strengths and Limitations section of the revised Discussion (page 25).

**Comments by reviewer #2**

**Comment 1:** The study is cross-sectional, but some interpretations suggest causality (e.g., “predicts pulmonary function decline”). My suggestion is to revise throughout to avoid causal language. Replace “predicts” with “is associated with” unless longitudinal data are included.

***Response:*** We appreciate this important clarification. All causal language (e.g., “predicts” or “pulmonary function decline”) has been systematically replaced with non-causal terminology such as “is associated with” and “lower pulmonary function” throughout the manuscript, including the title, abstract, results, discussion, and conclusion.

**Comment 2:** Discuss potential measurement error or variability and why BIA was chosen over DXA.

***Response:*** We have added justification for the use of bioelectrical impedance analysis (BIA), including references validating multi-frequency BIA against DXA in Korean populations. Potential measurement variability has also been acknowledged as a limitation in the revised manuscript.

These revisions are detailed in the Introduction (page 5) and Discussion section (page 25).

**Comment 3:** Physical activity, dietary factors, and socioeconomic status could confound both adiposity and pulmonary outcomes. Acknowledge these unmeasured confounders as a limitation.

***Response:*** Physical activity was partially addressed through inclusion of a regular exercise variable. However, dietary factors and socioeconomic status were not directly measured. These unmeasured confounders and their potential influence on both adiposity and pulmonary function are now explicitly acknowledged in the limitations section.

These revisions are detailed in the Discussion section (page 25).

**Comment 4:** Terms like “pulmonary function decline” imply longitudinal worsening; since this is cross-sectional, it should read “lower pulmonary function”.

***Response:*** All instances of “pulmonary function decline” have been replaced with “lower pulmonary function.”

**Comment 5:** Data collection time duration not mentioned.

***Response:*** The data collection period (January 2007 to December 2014) is now clearly stated in both the Abstract and Methods sections (page 2, 5).

**Comment 6:** Specify whether lung function was pre- or post-bronchodilator.

***Response:*** We clarified in the Methods section that all spirometry measurements were obtained prior to bronchodilator administration (page 7).

**Comment 7:** Add reference for BIA validation in Korean populations.

***Response:*** Appropriate references validating multi-frequency BIA in Korean adults have been added to the manuscript (page 5).

**Comment 8:** Clarify definition of “impaired pulmonary function” (thresholds used).

***Response:*** The Methods section has been revised to clearly define impaired pulmonary function using LLN-based criteria. Consistent with ATS/ERS recommendations for spirometry-only assessments, airflow limitation was defined as an FEV₁/FVC ratio < LLN, and a restrictive spirometric pattern was defined as FVC < LLN with a preserved FEV₁/FVC ratio. These definitions are now explicitly stated.

Relevant revisions were made in the Methods (page 7) and Results (page 19-21).

**Comment 9:** Figures are clear but could include 95% confidence intervals to improve interpretability.

***Response:*** Figure 1 has been updated to include 95% confidence intervals for FVC and FEV₁ across BMI categories and BF% quartiles.

**Additional Clarifications**

In accordance with the comprehensive revisions made throughout the manuscript, the Abstract has been updated to ensure consistency with the revised analyses and interpretations. The Conclusion has also been revised to reflect association rather than causation, integrate LLN-based findings, and emphasize key high-risk subgroups identified in the study.

We again thank the reviewers for their insightful and constructive comments. We believe that the revised manuscript now addresses all concerns and has been substantially strengthened in both methodological rigor and interpretative clarity.
